# Cognitive dysfunction following desflurane versus sevoflurane general anesthesia in elderly patients: a randomized controlled trial

**DOI:** 10.1186/2045-9912-4-6

**Published:** 2014-03-25

**Authors:** Minhthy Meineke, Richard L Applegate, Thomas Rasmussen, Donald Anderson, Sherif Azer, Ali Mehdizadeh, Amy Kim, Martin Allard

**Affiliations:** 1 Department of Anesthesiology, Loma Linda University School of Medicine, Room 2532 LLUMC, 11234 Anderson Street, Loma Linda, CA 92354, USA

**Keywords:** General anesthesia, Postoperative cognitive decline, Geriatric patient

## Abstract

As life expectancy increases, more patients ≥65 years undergo general anesthesia. Anesthetic agents may contribute to postoperative cognitive dysfunction, and incidence may differ with anesthetic agents or intraoperative anesthesia depth. Responses to anesthetic adjuvants vary among elderly patients. Processed electroencephalography guidance of anesthetic may better ensure equivalent cerebral suppression. This study investigates postoperative cognitive dysfunction differences in elderly patients given desflurane or sevoflurane using processed electroencephalography guidance.

IRB approved, randomized trial enrolled consenting patients ≥65 years scheduled for elective surgery requiring general anesthesia ≥120 minute duration. After written informed consent, patients were randomly assigned to sevoflurane or desflurane. No perioperative benzodiazepines were administered. Cognitive impairment was measured by an investigator blinded to group assignment using mini-Mental Status Examination (MMSE) at baseline; 1, 6, and 24 hours after the end of anesthesia. Mean arterial pressure was maintained within 20% of baseline. Anesthetic dose was adjusted to maintain moderate general anesthesia per processed electroencephalograpy (Patient State Index 25 to 50). The primary outcome measure was intergroup difference in MMSE change 1 hour after anesthesia (median; 95% confidence interval).

110 patients consented; 26 were not included for analysis (no general anesthesia; withdrew consent; baseline MMSE abnormality; inability to perform postoperative MMSE; data capture failure); 47 sevoflurane and 37 desflurane were analyzed. There were no significant differences in patient characteristics; intraoperative mean blood pressure (desflurane 86.4; 81.3 to 89.6 versus sevoflurane 82.5; 80.2 to 86.1 mmHg; p = 0.42) or Patient State Index (desflurane 41.9; 39.0 to 44.0 versus sevoflurane 41.0; 37.5 to 44.0; p = 0.60) despite a lower MAC fraction in desflurane (0.82; 0.77 to 0.86) versus sevoflurane (0.96; 0.91 to 1.03; p < 0.001). MMSE decreased 1 hour after anesthesia (p < 0.001). The decrease at one hour was larger in sevoflurane (−2.5; −3.3 to −1.8) than desflurane (−1.3; −2.2 to −0.5; p = 0.03). MMSE returned to baseline by 6 hours after anesthesia.

**Conclusions:**

For elderly patients in whom depth of anesthesia is maintained in the moderate range, both desflurane and sevoflurane are associated with transient decreases in cognitive function as measured by MMSE after anesthesia, with clinically insignificant differences between them in this setting.

**Trial registry:**

ClinicalTrials.gov NCT01199913

## Introduction

As life expectancy increases, an increasing portion of patients who undergo procedures involving general anesthesia are ≥65 years [1]. It is estimated that in 2012 the population >65 years represented 16% of the more developed nations [2], and by 2025 this age group will represent 21% of the industrialized population [3]. Postoperative cognitive decline or dysfunction (POCD) may occur after general anesthesia [4], and the risk of developing dementia in the subsequent 3 to 7 years following anesthesia and surgery is reported to be nearly doubled in elderly patients who develop POCD [5]. POCD at hospital discharge has been associated with greater mortality in the first 3 months and 1 year following surgery [4]. The type and severity of dysfunction may evolve over time [6]. Further, in patients >60 years, some types of postoperative cognitive dysfunction such as delirium may predict worsening cognitive function in the first year after surgery [7].

The cognitive sequelae after anesthesia with desflurane or sevoflurane deserve further investigation. Both agents have low blood-gas partition coefficients, allowing for shorter induction times compared to more soluble inhaled anesthetics. Some differences between the two agents have been reported, including a shorter emergence time following desflurane than sevoflurane anesthesia. While some investigators report similar POCD incidence [8], others have implicated sevoflurane in the development of mild cognitive impairment [9].

Anesthetic administration has been guided by use of age-adjusted estimations of minimum alveolar concentrations (MAC) [10,11], the concentration at which 50% of patients will not move in response to surgical incision [12]. MAC fraction is the ratio of anesthetic concentration divided by MAC for the specific agent, and is used to facilitate assessment of clinical differences between inhaled anesthetics [13]. The immobilizing effect of inhaled anesthetics is primarily mediated at the spinal cord level [14-16], although concentrations approaching 3 MAC will produce immobility when isolated brain anesthetic delivery is done in animals [17]. However, contemporary general anesthetic techniques include the use of neuromuscular blocking drugs that markedly reduce clinicians’ ability to use movement in response to incision as a guide to adequate anesthetic depth, and include addition of opioids or other anesthetic adjuvants to inhaled anesthetics. Increased sensitivity to drugs is common in elderly patients [18-20], and interindividual variability in drug responses results in differing sensitivity. These factors make predicting precise doses difficult [21], particularly for sedative, opioid and anesthetic drugs since physiologic function varies between patients of similar age [20,22]. It follows that when adjuvant drugs are added, the degree of cerebral suppression induced by controlling inhaled anesthesia depth using age-adjusted MAC could vary between patients.

Older patients given inhaled anesthetics require a lower MAC fraction to produce an equivalent degree of cerebral suppression measured by processed EEG [23]. If greater intraoperative cerebral suppression (deep anesthesia) is related to POCD, then assessing the impact of inhaled agents may be better done when equivalent degrees of cerebral suppression are maintained, rather than using equivalent estimations of anesthetic effect calculated from end-tidal anesthetic agent concentration. Several investigations have reported processed electroencephalogram (EEG) use to guide anesthesia administration in POCD study patients. Patients who had fewer episodes of deep anesthesia detected by processed EEG had less postoperative delirium [24], while patients in whom combined processed EEG and regional cerebral oximetry remained in moderate target ranges had less POCD [25]. Transient POCD was found in nearly half of elderly patients given desflurane or sevoflurane titrated to light general anesthesia as guided by processed (EEG) [26]. Of note, light general anesthesia may be associated with undesirable cardiovascular effects. This study was designed to investigate POCD differences between desflurane and sevoflurane in elderly patients in whom cerebral suppression is maintained in a moderate general anesthesia range guided by processed EEG.

## Methods

The Institutional Review Board of Loma Linda University approved this randomized controlled trial, which was registered in ClinicalTrials.gov (NCT01199913). Written informed consent was obtained from adult patients ≥65 years undergoing surgery requiring general anesthesia scheduled for ≥120 minutes. American Society of Anesthesiologists physical status and a measure of physiologic comorbidity (P-POSSUM) [27,28] were recorded. Patients were excluded for clinically significant cardiovascular, respiratory, hepatic, renal, neurological, psychiatric or metabolic disease. This included any prior history of cerebral vascular disease or dementia. Patients were also excluded if they weighed more than 150% of their ideal body weight (male: ideal body weight [in kg] = 50 + 2.3 kg per inch >5 feet; female ideal body weight [in kg] = 45.5 + 2.3 k g per inch >5 feet). To ensure ability to complete the cognitive function tests, patients who did not speak English or did not have at least an elementary school education were excluded from the study. Patients who had undergone a general anesthetic within the past 7 days were also excluded.

After consenting, patients were assigned to either desflurane or sevoflurane by computerized random sequence generator. Only the anesthesia provider was aware of the patient’s group assignment. Clinicians were allowed to reallocate group assignment if deemed clinically appropriate. The surgeons, nurses (operating room, recovery room and postoperative unit), patients and other investigators remained blinded to group assignment until study participation was completed. No inducement was offered for study participation. No preoperative sedation or perioperative benzodiazepine was given to patients. The risk of increased anxiety from withholding preoperative sedation was thoroughly discussed with patients during informed consent. Additionally, patients were informed they could request preoperative sedation and withdraw consent at any time prior to anesthesia induction, with subsequent exclusion from the study but no negative impact on their perioperative care.

Subjects received general anesthesia using propofol (2–2.5 mg/kg) for induction and the assigned inhaled anesthetic in oxygen and air for maintenance. Anesthesia delivery was titrated to the moderate general anesthesia range based on processed EEG (Patient State Index 25–50; SEDLine; Masimo, Irvine, CA, USA). Patients were excluded if data capture of the PSI values to the anesthesia record or internal storage drive on the processed EEG device failed. Mean arterial pressure was kept within 20% of patient’s baseline value as determined on the morning of surgery, with fluid and transfusion management decisions left to the discretion of the anesthesia provider. Ventilation was adjusted to maintain normocarbia (target end-tidal CO_2_ 35 to 40 mmHg) and total fresh gas flows were ≥ 2 l/min. The fraction of inspired oxygen and use of positive end expiratory pressure (PEEP) were determined by the anesthesia provider and adjusted as necessary to maintain adequate oxygenation (pulse oximeter saturation >92%). Intraoperative opioids were limited to fentanyl and hydromorphone. No morphine was administered to limit the risk for mental clouding from active morphine metabolites.

Patients were screened for cognitive impairment using the Mini-Mental Status Examination (MMSE) administered before and after the anesthetic. This screening test quantitatively assesses cognitive impairment on a scale from 0 to 30 (lower scores indicate worse impairment) based on answers to a variety of questions [29], and is suggested as one method to identify patients in whom cognitive impairment is suspected [30]. The MMSE has been reported to be a useful screening tool for POCD in hospitalized elderly patients [31], and may identify patients at greater risk for delirium following cardiac surgery [7]. Screening for cognitive impairment using the MMSE has been suggested as a way to identify patients in whom intervention could slow or halt the progression to dementia [4]. Use of the MMSE to evaluate patients after surgery under general anesthesia has been shown to be both easy and reliable [25,26,31]. A decrease in MMSE > 2 points was deemed clinically significant [32-34].

An investigator who was blinded to group assignment obtained MMSE prior to patient transfer to the operating room (baseline). MMSE was obtained postoperatively 1, 6, and 24 hours after the end of anesthesia. Patients were administered two versions of the MMSE, to ensure the patient did not take the same version at consecutive measurements to avoid a falsely elevated MMSE score as a consequence of learning from the previous MMSE. The same investigator who was blinded to group assignment obtained all MMSE for any patient. End of anesthesia was defined as the time when the inhaled agent was turned off and emergence time defined as the time between anesthesia end and tracheal extubation. We preferentially enrolled patients whose procedures were scheduled such that the MMSE 6 hours after anesthesia would not be obtained after 10 PM to minimize the impact of circadian rhythm on alertness. Patients were removed from study participation if the surgical procedure was not finished to allow administration of the MMSE 6 hours after anesthesia end prior to 10 PM as sleepiness can decrease MMSE score [35,36] and patients would be expected to be tired or sleepy at that time of day.

Verbal pain scores (ranging from 0 = no pain to 10 = most severe pain imaginable) were obtained at baseline and again before all MMSE administrations. Patients only received fentanyl or hydromorphone in the postanesthesia recovery room. The surgical team prescribed all analgesic medications after patients’ discharge from the recovery room. Opioids administered were converted to morphine equivalents. Postoperative nausea and vomiting was treated with ondansetron first, then if needed with metoclopramide. Management of patients who required further treatment for postoperative nausea and vomiting was at the discretion of the anesthesia provider, but patients who received sedative antiemetics such as promethazine prior to the MMSE one hour after anesthesia end were excluded from analysis.

Statistical methods: Sample size was calculated based on an intergroup difference of a least 2 points in MMSE decrease at one hour with power set to 0.8 and p = 0.05 considered statistically significant. Based on this calculation, 84 patients needed to successfully complete study participation to show statistical significance. Data analysis (JMP 10.0.0, SAS Institute, Cary, NC, USA) revealed continuous data were not normally distributed (Shapiro Wilk; all p < 0.05 indicated data were not normally distributed). Continuous data were analyzed by Wilcoxon test and expressed as median, 95% confidence interval. Repeated measurement data at the 4 measurement points for MMSE, test times and pain scores were analyzed by Friedman with Dunn’s multiple corrections test. Categorical data were analyzed by Chi square. For all analyses, p < 0.05 indicated statistical significance.

The primary outcome measure was the intergroup difference in MMSE change at one hour after anesthesia end. Secondary measures included intergroup differences in demographic characteristics; calculated age-adjusted MAC fraction based on end tidal anesthetic concentration [10], emergence time (minutes); PACU time (minutes); length of hospital stay (days); MMSE change over time; and MMSE change one hour after anesthesia compared to length of anesthesia.

## Results

**Figure 1:**
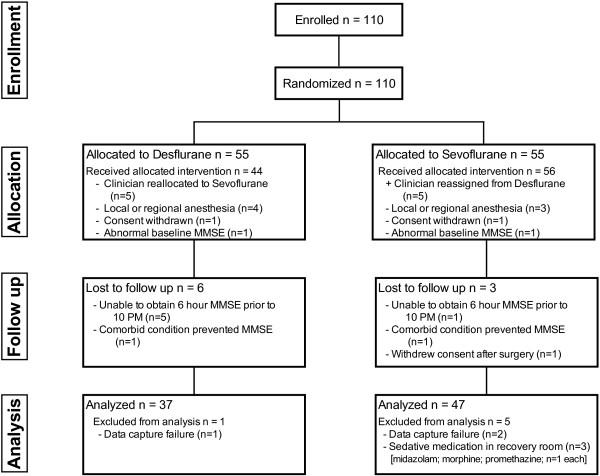
Consolidated Standards of Reporting Trials (CONSORT) flow diagram.

A total of 110 patients consented to participate and were randomized between September 2010 and January 2012, and 26 patients did not complete participation resulting in 84 patients completing study participation. Anesthesia providers changed group assignment resulting in 50 patients assigned to desflurane and 60 to sevoflurane. Several patients were lost to follow up (CONSORT diagram, Figure 1) leaving 37 desflurane and 47 sevoflurane patients for analysis. There were no intergroup differences in patient or perioperative characteristics (Table 1). The average PSI during surgery was within the target range in all patients, although 30 had PSI <25 for short periods of time, with no intergroup differences, and no patient had recorded PSI <25 for more than 10% of anesthesia time. The average calculated age-adjusted end-tidal anesthetic agent MAC fraction was lower in desflurane (0.82; 0.77 to 0.86 MAC) than sevoflurane (0.96; 0.91 to 1.03 MAC; p < 0.0001) to attain a similar average PSI (desflurane 41.9; 39.0 to 43.8 versus sevoflurane 41.0; 37.5 to 44.0; p = 0.60). The average age adjusted MAC did not correlate to the average PSI overall (R^2^ < 0.01) or in desflurane (p R^2^ = 0.05) or sevoflurane (R^2^ = 0.03) patients. All postoperative pain scores were higher than baseline regardless of group (p < 0.0001), but decreased from 1 hour to 6 and 24 hours after anesthesia end (p < 0.01). However, there were no significant intergroup differences in pain scores at any time, or in total opioid administration (all p > 0.05). There were no significant intergroup differences in other secondary outcome measures (Table 1). Regression analysis showed no relationship between the duration of anesthesia and the change in MMSE one hour after anesthesia end (R^2^ = 0.03).

**Table 1:** Patient and perioperative characteristics

	**Desflurane n = 37**	**Sevoflurane n = 47**	**p-value**
*Patient characteristics*			
Age years	72.3; 69.8 to 74.4	71.9; 70.1 to 73.6	0.96
Gender # female, male	24, 13	33, 14	0.60
Body mass index kg/m^2^	26.3; 25.3 to 27.6	26.6; 25.3 to 27.6	0.99
P-POSSUM physiology score	19.8; 17.4 to 21.7	19.1; 17.4 to 21.6	0.40
ASA physical status # 2, 3, 4	14, 23, 0	23, 22, 2	0.22
Comorbid conditions # (%) with			
Hypertension	26 (70.3%)	33 (70.2%)	0.99
Cardiovascular disease	5 (13.5%)	10 (21.3%)	0.36
Diabetes mellitus	7 (18.9%)	6 (12.8%)	0.44
Renal disease	4 (10.8%)	3 (6.4%)	0.47
Pulmonary disease	2 (5.4%)	8 (17.0%)	0.10
Preadmission medications # (%) taking			
Opioid	1 (2.7%)	0	0.26
Antihypertensive	26 (70.3%)	31 (66.0%)	0.67
Cardiac	3 (8.1%)	1 (2.1%)	0.20
Antihyperglycemic	6 (16.2%)	6 (12.8%)	0.65
Respiratory	1 (2.7%)	7 (14.9%)	0.06
Hypnotic	1 (2.7%)	0	0.26
Type of procedure performed # (%)			0.27
Intra-abdominal	3 (8.1%)	4 (8.5%)	
Other general surgery	1 (2.7%)	3 (6.4%)	
Pelvic (gynecologic or urologic)	27 (73.0%)	25 (53.2%)	
Orthopedic	6 (16.2%)	15 (31.9%)	
*Intraoperative characteristics*			
Average patient state index	41.9; 39.0 to 43.8	41.0; 37.5 to 44.0	0.60
Average age adjusted anesthetic minimum alveolar concentration fraction%	0.82; 0.77 to 0.86	0.96; 0.91 to 1.03	<0.0001
Mean arterial blood pressure mmHg	86.4; 81.3 to 89.6	82.5; 80.2 to 86.1	0.42
Mean arterial blood pressure change from preoperative baseline blood pressure %	−5.7; −10.7 to −0.7%	−9.2; −14.0 to −4.9%	0.18
End tidal carbon dioxide mmHg	33.6; 33.1 to 34.6	33.2; 32.7 to 33.6	0.28
Pulse oxygen saturation %	98.4; 98.0 to 99.0	98.6; 9.1 to 99.0	0.67
Anesthesia time minutes	144; 119 to 170	139; 125 to 157	0.89
Surgery time minutes	118; 92 to 148	119; 108 to 133	0.60
Emergence time minutes	7.7; 6.3 to 9.1	8.2; 6.9 to 9.6	0.51
Cough on tracheal extubation # (%) yes	8 (21.6%)	12 (25.5%)	0.68
Opioid morphine equivalents mcg/kg/hour	116.1; 91.1 to 151.2	143.2; 110.3 to 165.4	0.35
*Postoperative Characteristics*			
Recovery room opioids morphine equivalents mcg/kg	99.3; 54.7 to 144.6	101.6; 78.9 to 145.6	0.44
Postoperative nausea / vomiting # yes	18 (48.7%)	17 (36.2%)	0.25
Recovery room length of stay minutes	105; 96 to 114	102; 91 to 114	0.74
Hospital length of stay, days	1.6; 1.1 to 2.0	1.4; 1.2 to 2.0	0.63

Table 1: There were no significant intergroup differences in patient and perioperative characteristics of 84 patients anesthetized with either desflurane or sevoflurane. Continuous data were not normally distributed (Shapiro Wilk p < 0.05) and were expressed as median, 95% confidence interval and analyzed by Wilcoxon. Categorical data were analyzed by Chi square.

**Figure 2:**
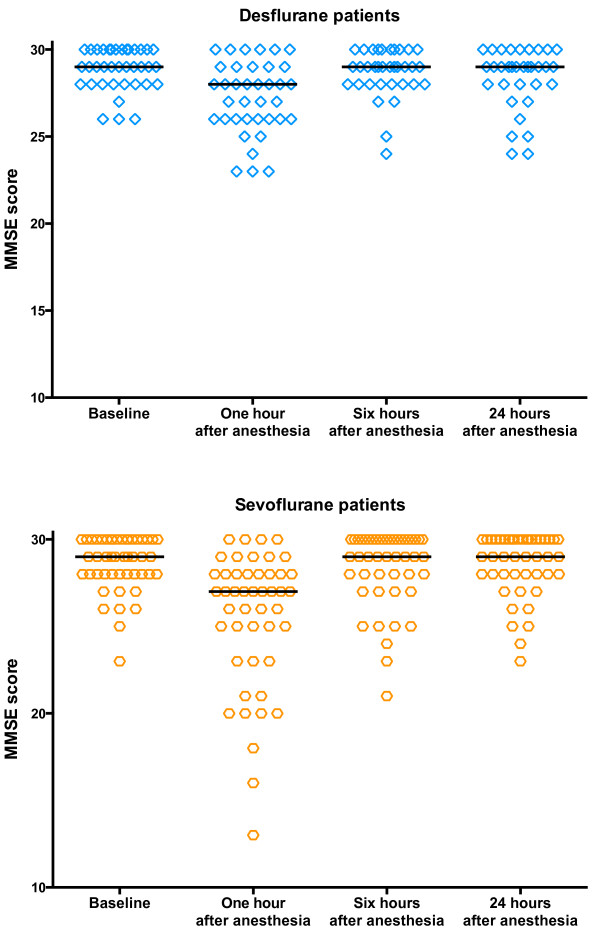
**Comparison of Mini Mental State Exam (MMSE) scores in patients given desflurane (n = 37) or sevoflurane (n = 47) anesthesia, numbers indicate median and 95% confidence interval.** The MMSE was lower one hour after anesthesia for both groups (p < 0.001 Wilcoxon matched pairs). The MMSE scores were equal to baseline at 6 and 24 hours after the end of anesthesia, and there were no intergroup differences in MMSE scores at any measurement time (all p > 0.05, Friedman with Dunn’s multiple corrections test).

**Figure 3:**
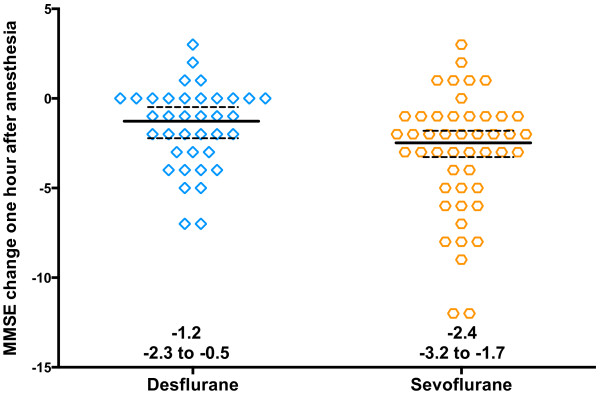
**Change in Mini Mental Status Exam (MMSE) score one hour after the end of anesthesia, numbers indicate median, 95% confidence interval.** Although the MMSE score decreased more in the 47 patients anesthetized with sevoflurane (−2.4, −3.2 to −1.7) than in the 37 patients anesthetized with desflurane (−1.3; −2.3 to −0.5; p = 0.04 Wilcoxon), the difference in the magnitude of decrease was not clinically significant (not at least 2 points).

There were no significant intergroup differences in MMSE scores at any time point. MMSE one hour after anesthesia decreased from baseline in both sevoflurane and desflurane (p < 0.001; Figure 2). While the magnitude of decrease was small, the change in MMSE one hour after anesthesia was greater in sevoflurane (−2.5; −3.3 to −1.8) than desflurane (−1.3; −2.2 to −0.5; p = 0.03; Figure 3), but the difference between groups was not clinically significant (not at least 2 points). MMSE one hour after anesthesia was more likely to decrease from baseline in sevoflurane (85.1%) than desflurane (62.2%; p = 0.02), and clinically significant (at least 2 point) decrease was more likely in sevoflurane (68.1%) and desflurane (46.0%; p = 0.04; Table 2). Three patients were discharged prior to obtaining the MMSE 6 hours after anesthesia and an additional 3 were discharged prior to obtaining the MMSE 24 hours after anesthesia. Analysis of the remaining patients showed that MMSE was not significantly different either between groups or from baseline at 6 or 24 hours. Testing time increased one hour after anesthesia (6.6; 5.9 to 7.5 minutes) compared to baseline (5.1; 4.9 to 5.3 minutes; p < 0.0001), but was the same as baseline 6 and 24 hours after anesthesia. Testing time was not different between groups at any point.

**Table 2:** Comparison of mental status examination scores

	**Desflurane n = 37**	**Sevoflurane n = 47**	**p-value**
MMSE baseline (n = 84)	29.1	29.0	0.63
	28.6 to 29.5	28.3 to 29.5	
MMSE 1 hour after anesthesia (n = 84)	27.5	26.7	0.07
	26.5 to 28.3	25.6 to 27.4	
Change in MMSE at 1 hour (n = 84)	−1.3	−2.5	0.03
	−2.2 to −0.5	−3.3 to −1.8	
MMSE decreased at 1 hour # (%) yes	23 (62.2%)	40 (85.1%)	0.02
MMSE decrease at least 2 points 1 hour after anesthesia end # (%) yes	17 (46.0%)	32 (68.1%)	0.04
MMSE 6 hours after anesthesia (n = 81)	28.9	29.2	0.70
	28.5 to 29.4	28.5 to 29.7	
MMSE 24 hours after anesthesia (n = 78)	28.9	29.2	0.39
	28.3 to 29.3	28.3 to 29.7	

Table 2: Mini Mental Status Exam (MMSE) scores in patients anesthetized with sevoflurane or desflurane. Continuous data are presented as median, 95% confidence interval and were analyzed by Wilcoxon; categorical data were analyzed by Chi square. Three patients were discharged prior to obtaining the MMSE 6 hours after anesthesia and an additional 3 were discharged prior to obtaining the MMSE 24 hours after anesthesia. The MMSE was not significantly different between groups at any measurement time. While the MMSE obtained one hour after anesthesia end was more likely to decrease from baseline in sevoflurane than desflurane, this difference was less than considered clinically significant (not at least 2 points) and was not present at 6 or 24 hours after anesthesia.

## Discussion

We were not able to demonstrate a clinically significant (at least 2 point) difference in MMSE one hour after anesthesia end in patients ≥65 years given sevoflurane compared to desflurane titrated to moderate general anesthesia guided by processed EEG (PSI 25 to 50). Despite the slightly greater likelihood of MMSE decrease one hour after anesthesia in sevoflurane, we were not able to show an intergroup difference of at least 2 points. Further, the small decrease found at one hour was no longer present and MMSE had returned to baseline by 6 hours after anesthesia. Thus, our results show only a minimal transient decrease in cognitive function assessed by MMSE one hour after anesthesia with no clinically significant difference between sevoflurane and desflurane when administered as in this setting.

These findings are similar to those found in elderly patients in whom the inhalation agent was titrated to light general anesthesia guided by processed EEG (bispectral index 55 to 65) [26]. However, the average MMSE decrease reported in that study was <2 points for both sevoflurane and desflurane, and the proportion who had at least 2 point decrease was not reported. A subgroup analysis revealed more POCD when patients had more intraoperative episodes of deep anesthesia indicated by processed EEG monitoring [24]. POCD was less frequent in elderly patients when moderate anesthesia depth was maintained using processed EEG guidance compared to routine care based on clinical signs and end-tidal anesthetic agent concentration [37]. The use of processed EEG guidance was associated with less anesthetic agent delivery, but no comparison of inhaled agents was provided. Studies in which anesthesia administration was guided by inspired or end-tidal anesthetic concentration and age-adjusted estimations of minimum alveolar concentrations (MAC) could be confounded by the impact of drug co-administration. The presence of interindividual variability in response to opioids and the variable effects of aging on pharmacodynamics, suggest that titrating anesthesia depth based solely on age-adjusted MAC could result in different degrees of cerebral suppression. We titrated inhaled anesthetics to the processed EEG value and found a lower MAC fraction in desflurane than sevoflurane. Although this study was not designed to investigate the relationship between end-tidal anesthetic agent concentration and processed EEG values, the lower MAC fraction in desflurane is consistent with studies demonstrating lower processed EEG values at 1 MAC in desflurane compared to sevoflurane anesthetized patients [38]. Thus titration to similar processed EEG values would be expected to require a lower MAC fraction for desflurane than sevoflurane. Assessing the contribution of anesthetic agents to postoperative cognitive impairment could thus be confounded by greater intraoperative cerebral suppression (deep anesthesia) at the calculated age-adjusted MAC. It would seem prudent to include equivalent degrees of cerebral suppression rather than estimations of anesthetic effect when comparing postoperative cognitive effects of specific anesthetic agents.

Several factors limit generalization of our findings to other settings. Importantly, fewer patients received desflurane than sevoflurane based on changing group assignment by individual clinical anesthesiologists, typically for concerns about airway reactivity. Reallocation was allowed in the protocol as our research community considered this to be important for patient safety. It is possible that the clinician could have been subtly influenced to choose one agent over the other, but despite this we found no significant differences in patient or perioperative characteristics. The clinical anesthesiologists did not obtain MMSE scores for any patient. Further, we found no intergroup differences in intraoperative opioid administration but did find similar depth of anesthesia as measured by processed EEG. Thus it is unlikely that patient reallocation by clinicians significantly impacted any differences in MMSE. Another limitation is that pain increased from baseline to the time MMSE was obtained one hour after anesthesia end. It is possible that increased pain itself contributed to lower MMSE scores at that time. However, we did not find intergroup differences in either verbal pain scores or the change in pain score from baseline at this or other times that MMSE was obtained and opioid administration was similar in the groups. Thus while the decrease in MMSE one hour after anesthesia end may have been partly caused by increased pain, the slightly greater magnitude of MMSE decrease in sevoflurane is not likely based solely on pain. We chose to perform analysis based on the agents as administered since we were concerned about the clinical effects of the agents.

We used the MMSE to screen for cognitive dysfunction instead of more complete testing panels as some have used [39-41], which may also limit generalization of our findings. While the MMSE may require more time to complete than some consider ideal, it can detect cognitive impairment in elderly patients, as suggested ideal for preoperative screening [42]. Additionally the MMSE has been used in a wide range of clinical settings, including in elderly surgical patients [25,26,31], which makes it a reasonable screening tool. The greater sensitivity of more extensive testing panels is associated with a longer test time, which elderly patients may not be willing to perform in the immediate postoperative period. The MMSE has high specificity for detecting mild cognitive impairment [43], and was thus chosen for use in our study setting. Six of the 9 patients lost to follow up (Figure 1) did not complete study participation based on surgery taking longer than scheduled, which would have resulted in MMSE testing at late times of the day. Removal of these patients could have impacted the small intergroup difference we found at one hour. The choice to not complete the study on these patients was deemed appropriate since sleepiness has been associated with lower MMSE score [35,36]. Several patients were discharged prior to obtaining the MMSE at 6 and 24 hours after anesthesia, which could confound comparisons at those times, although it is likely that patients deemed ready for discharge would have MMSE similar to their baseline. Exclusion of these 6 patients did not alter results for the intergroup difference in MMSE change from baseline one hour after anesthesia. Only 9 of our patients were ≥80 years old. This small number prevents us from detecting any difference between sevoflurane and desflurane in very old patients. Few patients had significant duration of low PSI, which limits our ability to assess a possible impact of deep anesthesia on change in MMSE. This is to be expected since the protocol specified titration of inhaled agents to moderate general anesthesia as guided by processed EEG. However, our findings suggest processed EEG monitoring can be successfully used to guide titration of inhaled anesthetic dosing in the elderly.

## Conclusions

In patients ≥65 years old, administration of desflurane or sevoflurane titrated to moderate general anesthesia as guided by processed EEG (PSI 25 to 50) was not associated with a lasting decrease in MMSE of at least 2 points. Our finding of only a slightly larger transient decrease in cognitive performance following sevoflurane compared to desflurane suggests either may be acceptable for geriatric patients when titrated to moderate general anesthesia. Further research is warranted to determine if the effects of these agents would differ when titrated to deep general anesthesia as indicated by processed EEG.

## Competing interests

All authors declare that they have no competing interests to disclose

## Authors’ contributions

MM participated in subject enrollment, data collection, data analysis and manuscript preparation; RA participated in study design, data analysis, and manuscript preparation; TR participated in study design, subject enrollment and data collection; DA, SA and AM participated in subject enrollment and data collection; AK participated in study design; MA participated in study design and manuscript preparation. All authors read and approved the final manuscript.
